# Comparative Transcriptome Analysis of the Skin and the Peritoneal Wall Layer of *Triplophysa stenura* Distributed in High Elevations

**DOI:** 10.3390/biology13010005

**Published:** 2023-12-20

**Authors:** Li Ma, Zhen Zhu, Shanzhong Zhang, Ruibin Yang, Chen Liu, Yongyao Yu, Xuefen Yang

**Affiliations:** 1 College of Fisheries, Huazhong Agricultural University, Wuhan 430070, China; mali19980308@163.com (L.M.); zhuzhen202009@163.com (Z.Z.); rbyang@mail.hzau.edu.cn (R.Y.); liuchen111@webmail.hzau.edu.cn (C.L.); yuyy@mail.hzau.edu.cn (Y.Y.); 2 Hubei Vocational College of Bio-Technology, Wuhan 430070, China; 3 Hechuan Campus, Sichuan Fisheries School, Hechuan, Chongqing 401520, China; zhangshanzhong1491@163.com

**Keywords:** transcriptome, skin, peritoneal wall layer, DEGs, melanin synthesis, *Triplophysa stenura*

## Abstract

**Simple Summary:**

Strong ultraviolet radiation is an extreme environmental characteristic of the Tibetan Plateau. Melanin is the most important light-protective substance that allows fish to resist ultraviolet radiation. In order to explore the melanin protection mechanism of fish in the Tibetan Plateau against strong ultraviolet radiation, *Triplophysa stenura* distributed in high elevation areas was selected as the research object. Transcriptome differences were compared between skin and peritoneal wall layers with different melanin contents to explore the regulatory genes related to melanin synthesis. This provides basic data for analyzing the molecular mechanism of melanin protection in plateau fish against strong ultraviolet radiation. The results indicate that a total of twenty-three DEGs are enriched in the melanin synthesis pathway by a local Blast comparison, of which nine DEGs are significantly upregulated in the peritoneal wall layer and six DEGs are significantly upregulated in the dorsal and lateral skin. The results suggest that these genes may be associated with the molecular mechanism of melanin synthesis in *T. stenura*, and the differential regulation of genes may be related to the differences in the UVR intensity and tissue sites of melanin synthesis. Further investigation is needed to determine how these genes specifically regulate melanin synthesis.

**Abstract:**

A total of 81,868 All-Unigenes were sequenced and assembled by the transcriptome in the dorsal skin, the lateral skin, and the peritoneal wall layer of *Triplophysa stenura* with a total assembly length of 123,827,585 bp, and 68,750 unigenes were annotated to seven functional databases. A total of 588 DEGs were screened between the dorsal and lateral skin, 17,097 DEGs were screened between the dorsal skin and the peritoneal wall layer, and 16,598 DEGs were screened between the lateral skin and the peritoneal wall layer. Most of DEGs in three tissues were annotated to GO terms related to cellular structures, binding, cellular processes, and catalytic activity. They were also annotated to KEGG pathways such as the MAPK signaling pathway, PI3K-Akt signaling pathway, Wnt signaling pathway, melanogenesis, tyrosine metabolism, and cell cycle. A total of twenty-three DEGs were found to be enriched in the melanin synthesis pathway by a local Blast comparison, of which nine DEGs were significantly upregulated in the peritoneal wall layer and six DEGs were significantly upregulated in the dorsal and lateral skin. The results suggest that these genes may be associated with the molecular mechanism of melanin synthesis in *T. stenura*, and the differential regulation of genes may be related to the differences in UVR intensity and tissue sites of melanin synthesis. Further investigation is needed on how these genes specifically regulate melanin synthesis.

## 1. Introduction

Melanin is the most important light-protective substance in fish. It can protect the skin and the internal organs from damage caused by ultraviolet radiation (UVR) [1,2,3,4]. During the evolutionary process, fish can adapt to the ultraviolet radiation environments by changing the number and content of melanophores [5].

Currently, research on the molecular regulatory mechanisms of fish melanophore proliferation and melanin synthesis mainly employs two methods: one is to study the molecular regulatory mechanisms of pigment generation by performing pure homozygous mutations on zebrafish (*Danio rerio*) and *Oryzias latipes* [6,7,8,9]; the other is to screen candidate genes related to pigment generation through a comparative transcriptomics analysis of fish with different body color phenotypes [10,11].

For now, there are 325 pigment-synthesis-related genes reported only in zebrafish, involving 26 functional proteins, indicating that the mechanism affecting pigment synthesis is complex [12]. Among them, the regulatory pathways involved in black melanin synthesis include α-MSH/MC1R, Wnt/β-catenin, Scf/c-Kit, and ET-1/ETB-R.

The ultraviolet radiation (UVR) in the Qinghai–Tibet Plateau is stronger due to its geographical and meteorological conditions. The water in the Qinghai–Tibet Plateau is clear with few particles and organic matter blocking the UVR, which makes it easier for UVR to penetrate into deeper waters and have a more severe impact on fish [13]. High UVR plays an important role in high-altitude adaptations of fish [14].

According to previous research, the melanin content of the skin and the peritoneum of *T. stenura* is higher at high altitudes than that at low altitudes. In addition, the outer membranes of important organs in fish also contain melanin, which has been preliminarily proven to play an important role in the resistance of high-altitude fish to UVR damage. However, the molecular mechanism of melanin generation in high-altitude fish adapting to strong UV radiation on the Qinghai–Tibet Plateau is currently unclear, except for a study that showed that the differential expression genes of the skin and peritoneal wall layer of *Schizopygopsis younghusbandi* at different altitudes are related to tyrosine metabolism and melanin synthesis [15].

The *Triplophysa* genus of fish is the most widely distributed fish on the Qinghai–Tibet Plateau, with its distribution ranging from altitudes of 100 m to 5000 m [16]. *T. stenura*, a type of high-altitude fish, is ideal for studying the biological effects of strong ultraviolet radiation on the plateau [17]. The skin and melanin distribution characteristics of *T. stenura* show that the number of melanophores on the dorsal skin is the highest, and the melanin content of the peritoneal wall layer is the highest [18]. Melanophore development and melanin production in different tissues may have different regulatory mechanisms.

This study aims to conduct a transcriptome analysis on the skin and the peritoneum of *T. stenura*, which is distributed in the Lhasa section of the Lhasa River, to identify key candidate genes that regulate melanin synthesis in the skin and the peritoneum of *T. stenura*. The study aims to explore the molecular mechanism by which *T. stenura* individuals regulate melanin synthesis to protect their bodies from UV radiation damage during adaptive evolution in response to strong UV radiation environments on the Qinghai–Tibet Plateau. The results of this study provide scientific data for the protection of fish from UVR damage on the Qinghai–Tibet Plateau.

## 2. Materials and Methods

### 2.1. Fish Samples

We collected samples of *T. stenura* from the Lhasa section of the Lhasa River at an altitude of approximately 3600 m in December 2020. We chose fresh and healthy fish for the experiment. The average body length of the fish was 8.21 ± 0.62 cm, and the weight was 6.19 ± 0.85 g.

Three replicates were analyzed. The fish were anesthetized using MS-222 (200 mg/L) (Solarbio, Beijing, China) with sample from the same location, cut off on the dorsal skin (D), the lateral skin (L), and the peritoneal wall layer (P) tissues of each fish. The samples were placed in an enzyme-free sterile cryopreservation tube and cooled in a liquid nitrogen tank.

All the methods were carried out in accordance with approved guidelines by the Animal Care and Ethic Committee of Huazhong Agricultural University.

### 2.2. Experimental Methods

#### 2.2.1. Total RNA Extraction

Total RNA was obtained from skin and peritoneum samples of *T. stenura* using RNAiso Plus Reagent (TaKaRa, Shiga, Japan) according to the manufacturer’s protocol. Total RNA was extracted with a Qubit^®^ RNA Assay Kit in a Qubit^®^ 2.0 Fluorometer (Life Technologies, Carlsbad, CA, USA). The RNA Nano 6000 Assay Kit from the Agilent Bioanalyzer 2100 system (Agilent Technologies, Santa Clara, CA, USA) and gel electrophoresis was used to assess the quantity and quality of the total RNA.

#### 2.2.2. RNA-Seq, De Novo Transcriptome Assembly

We used the filtering software SOAPnuke (v1.5.2), which was independently developed by BGI Biotechnology, for filtering to remove reads containing connectors (connector contamination) and reads with an unknown base N content of greater than 5% and to remove low-quality reads (defined as reads with a mass value below 15, accounting for more than 20% of the total base number of reads). The filtered ‘Clean Reads’ were saved in FASTQ format.

We used Trinity (v2.15.1) to perform de novo assembly on clean reads and Tgicl (v2.1) to cluster and remove redundancies from the assembled transcripts to obtain the Unigene. For multiple samples, we used the Tgicl again to cluster the Unigene of each sample to remove redundancies and obtain the final Unigene for subsequent analysis.

#### 2.2.3. Annotation of the Transcriptome and Functional Enrichment Analysis

The assembled Unigene and KEGG databases (Kyoto Encyclopedia of Gene and Genome), GO databases (Gene Ontology), NR databases (NCBI non-redundant protein databases), NT databases (non-redundant nucleic acid sequence databases), SwissProt databases (protein databases), Pfam databases (protein family databases), and KOG databases were annotated for Blastx (v2.14.1) comparison (E-value < 10^−5^).

Based on annotation information such as NR, genes were classified into GO functional categories. After obtaining GO annotations for Unigenes using Blast 2 GO software (v2.5), all Unigenes were statistically classified into GO functional categories using WEGO software (v2.0) in order to gain a macroscopic understanding of the functional distribution characteristics of DEGs. We analyzed the similarity of Unigene sequences with the KOG database and KEGG database through Blastx and identified the relevant biological pathways involved in Unigenes through database annotation information.

#### 2.2.4. Gene Expression Level Analysis

We used Bowtie2 (v2.2.5) to compare clean reads to the reference sequence to calculate the gene alignment rate and then used RSEM (v1.2.12) to calculate the expression levels of genes and transcripts to obtain the FPKM values. According to the set expression threshold, genes greater than this threshold participate in Venn plot calculation.

#### 2.2.5. Differential Gene Expression Analysis

We used the DESeq2 algorithm for differential gene detection. Differential expression genes (DEGs) were detected using the method described by [19,20] with Q-values (Adjusted *p*-value) ≤ 0.05. We used a difference multiple of two or more and a corrected *p* ≤ 0.05 to screen for DEGs. Based on the results of differential gene detection, we used the *p*-heatmap function in R software (v4.2.2) for the hierarchical clustering analysis. When multiple sets of DEGs were clustered simultaneously, a cluster analysis was conducted separately for the intersection DEGs and the union DEGs between groups. Based on the GO annotation results and official classification, differential genes were functionally classified and enriched using the phyper function in R software. The calculation method for *p*-values is as follows:

p=1−∑i=0m−1(Mi)(N−Mn−i)(Nn)

Then, we performed the FDR correction on the *p*-values, and typically, functions with FDR ≤ 0.01 were considered to be significantly enriched. Based on the KEGG annotation results and official classification, differential genes were classified into biological pathways, and an enrichment analysis was also performed using the phyper function in R software.

#### 2.2.6. Validation of Differential Gene Expression by qRT-PCR

Using fluorescence quantitative PCR to validate the transcriptome data results, seven genes were randomly selected from the DEGs, and α-Tublin was used as an internal reference gene. The first-strand cDNA was obtained from the total RNA using random primers and MMLV reverse transcriptase (Promega, Madison, WI, USA). The primers were designed using Beacon Designer (v8.14) with three technical replicates and three experimental replicates set for each gene during the experimental process. qRT-PCR was performed using SYBR Green PCR Super Mix (Thermo Scientific, Wilmington, DE, USA) and the CFX96 real-time PCR detection system (Bio Rad, Hercules, CA, USA) [9].

The 20 µL reaction system conducted by PCR includes 4 μL 5× Reaction Buffer, 0.5 μL Oligo (dT) 18 Primer (100 μM), 0.5 μL Random Hexamer primer (100 μM), 1 μL Servicebio^®^ RT Enzyme Mix (Servicebio, Wuhan, China), 10 μL Total RNA, and 20 μL RNase-free water. The expression level of the target gene was determined by α-Tublin standardization using log_2_ (fold change) to calculate the difference in gene expression between different tissues of *T. stenura* [21].

## 3. Results

### 3.1. Sequencing and Assembly of the T. stenura Transcriptome

The dorsal skin, the lateral skin, and the peritoneal wall layer tissues of *T. stenura* were sequenced using DNBSEQ platform technology to construct nine sample libraries, and after filtering raw reads data, 43.22 M, 43.57 M, 42.13 M, 38.09 M, 42.74 M, 42.17 M, 42.47 M, 42.07 M, and 37.75 M clean reads were obtained for each sample library.

**Figure 1:**
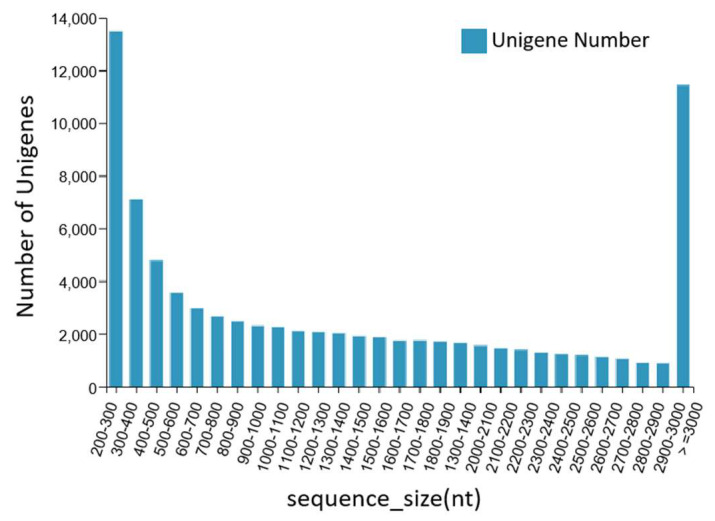
Length distribution information of the Unigenes.

The DNBSEQ platform was used to sequence the dorsal skin, the lateral skin, and the peritoneal wall layer tissues of *T. stenura*. A total of 56.13 Gb of data was obtained, and the basic Unigene information was obtained by assembling the results. After clustering assembly, there were a total of 81,868 All-Unigenes with a total length of 123,827,585 bp, an N50 of 2514 bp, and a GC content of 43.05%. The distribution information showed that Unigenes contained 200 bp or more, with 13,497 Unigenes being between 200 and 300 bp and 11,447 Unigenes being greater than or equal to 3000 bp. The data assembly integrity was relatively high (Figure 1).

**Figure 2:**
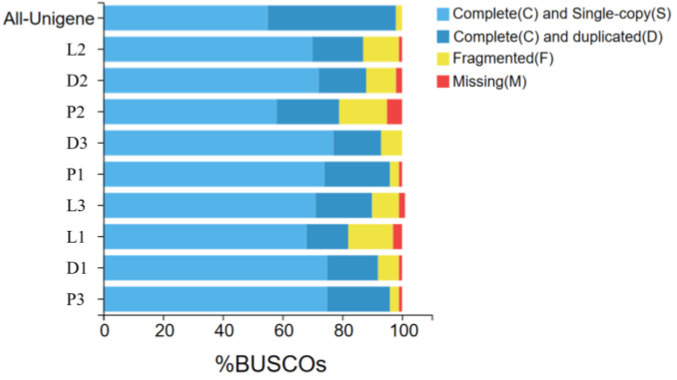
Results of the assembly evaluation of the BUSCO database.

According to the assembly evaluation results (Figure 2), 97% of the assembled transcripts matched all or part of the BUSCO database, with only the second sample of the peritoneal wall layer showing a 95% match to the BUSCO database. This indicates that the data from the different tissue sequencing assemblies of *T. stenura* are relatively neat and can be used for further analysis.

### 3.2. Annotation and Functional Classification

**Figure 3:**
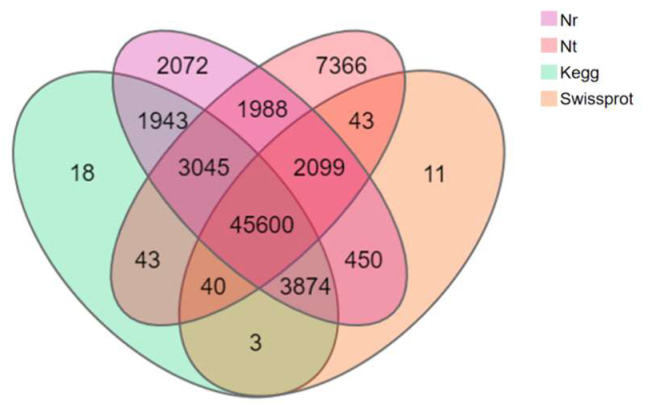
Venn diagram of the annotation results of the Unigene sequence against four databases.

A total of 28,800 (35.18%) of the 81,868 Unigenes of *T. stenura* were annotated in all databases simultaneously, while 68,750 Unigenes (83.98%) were annotated in one of the databases. Among the 68,750 annotated Unigenes, 45,600 (66.33%) were simultaneously annotated in all four major databases (Figure 3).

A total of 45,576 Unigenes assembled from the sequencing of the dorsal skin, the lateral skin, and the peritoneal wall layer tissues of *T. stenura* were annotated to the KOG database and clustered into 25 functional categories. 

**Figure 4:**
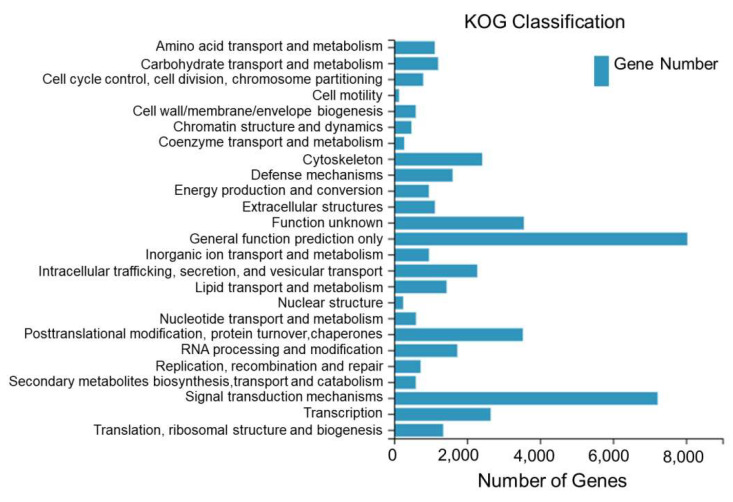
KOG classification of Unigenes in different tissues of *T. stenura*.

From Figure 4, it can be seen that 8035 Unigenes are clustered into general functional predictions, 7221 Unigenes are clustered into signal transduction mechanisms, 3556 Unigenes are clustered into unknown functions, 3528 Unigenes are clustered into post-translational modifications, protein folding, and chaperones, 2645 Unigenes are clustered into transcription, 2414 Unigenes are clustered into the cell skeleton, and the remaining 18,177 Unigenes are clustered into other 19 functional categories.

**Figure 5:**
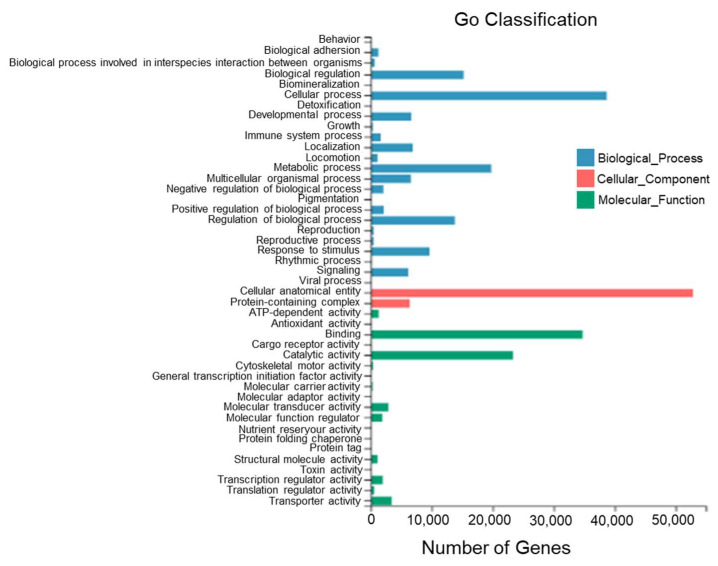
GO functional classifications of Unigenes in different tissues of *T. stenura*.

According to the sequence homology, all Unigenes of *T. stenura* were annotated to the GO database (Figure 5). The GO entries annotated to the biological process category were mainly clustered into cellular processes (38,694 Unigenes), metabolic processes (19,751 Unigenes), biological regulation (15,209 Unigenes), and the regulation of biological processes (13,787 Unigenes).

**Figure 6:**
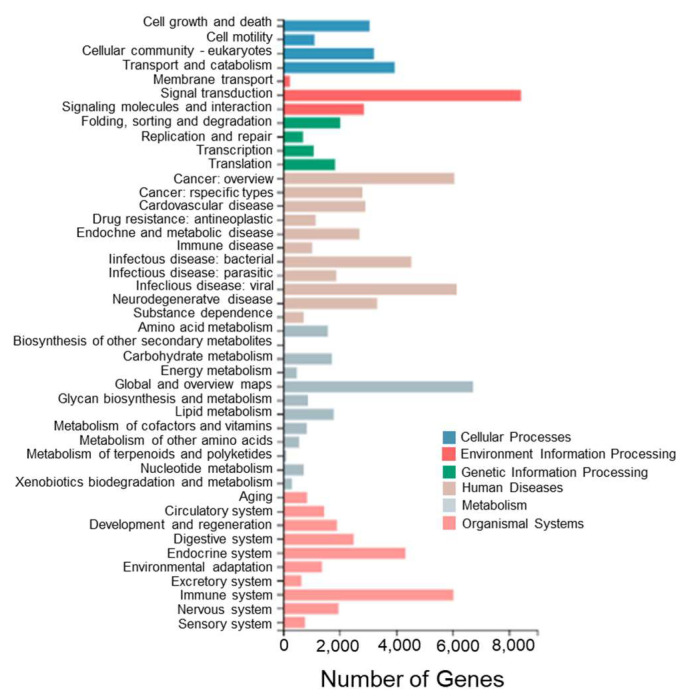
KEGG functional classifications of Unigenes in different tissues of *T. stenura*.

To further investigate the biological pathways between different tissues of *T. stenura*, a pathway enrichment analysis of Unigenes in the transcriptome was performed. The Unigenes were annotated to the KEGG database and classified into different functional categories at level 1 and level 2 (Figure 6).

Among these enriched pathways, the ones with the most enriched genes are signal transduction (8430 Unigenes), global and overview maps (6730 Unigenes), viral infectious diseases (6157 Unigenes), cancer overview (6065 Unigenes), immune system (6034 Unigenes), bacterial infectious diseases (4539 Unigenes), the endocrine system (4338 Unigenes), transport and catabolism (3954 Unigenes), neurodegenerative diseases (3331 Unigenes), and eukaryotic cell communities (3218 Unigenes).

### 3.3. Screening of DEGs in the Transcriptome in Different Tissues

**Figure 7:**
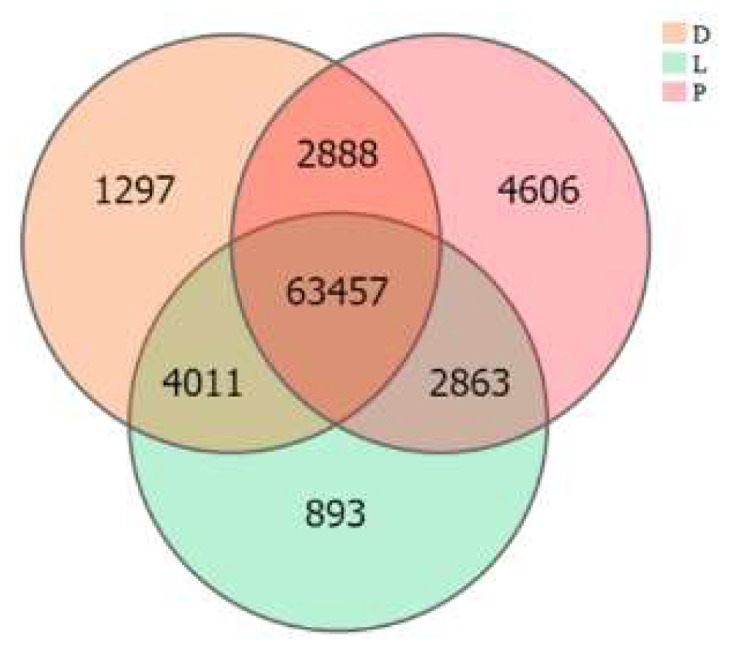
Gene expression in different tissues of *T. stenura* in a Venn diagram.

The inter-group expression of the dorsal skin, the lateral skin, and the peritoneal wall layer tissues of *T. stenura* showed that 63,457 genes are expressed simultaneously in all three tissues (Figure 7). The peritoneal wall layer expressed the most genes, while the lateral skin expressed the least.

**Figure 8:**
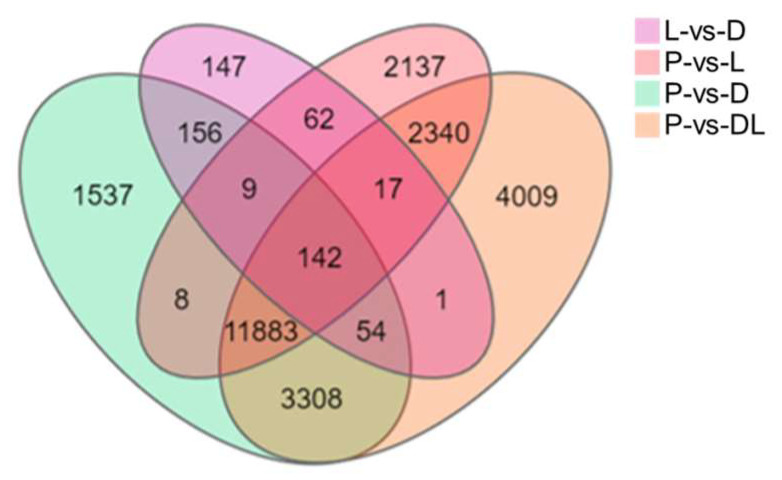
Differentially expressed genes in different tissues of *T. stenura* in a Venn diagram.

**Figure 9:**
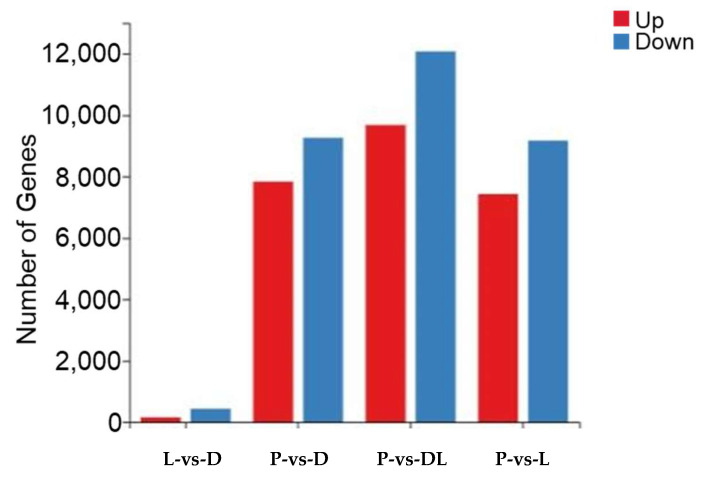
Differentially expressed genes in different tissues of *T. stenura* in a bar chart.

By analyzing the transcriptome results of the dorsal skin, the lateral skin, and the peritoneal wall layer of *T. stenura*, DEGs were obtained for different comparison groups (Figure 8). The gene expression levels of each sample were analyzed, and it was found that most genes were upregulated or downregulated (Figure 9).

The results indicate that 588 DEGs were screened between the dorsal and lateral skin. Compared to the lateral skin, there were 156 upregulated DEGs and 432 downregulated DEGs in the dorsal skin. Between the dorsal skin and the peritoneal wall layer, 17,097 DEGs were screened. Compared to the peritoneal wall layer, there were 7834 upregulated DEGs and 9263 downregulated DEGs in the dorsal skin. Between the lateral skin and the peritoneal wall layer, 16,598 DEGs were screened. Compared to the peritoneal wall layer, there were 7431 upregulated DEGs and 9167 downregulated DEGs in the lateral skin. Additionally, compared to the peritoneal wall layer, there were a total of 9677 upregulated DEGs and 12,077 downregulated DEGs in both the dorsal and lateral skin.

### 3.4. GO Annotation of DEGs

**Figure 10:**
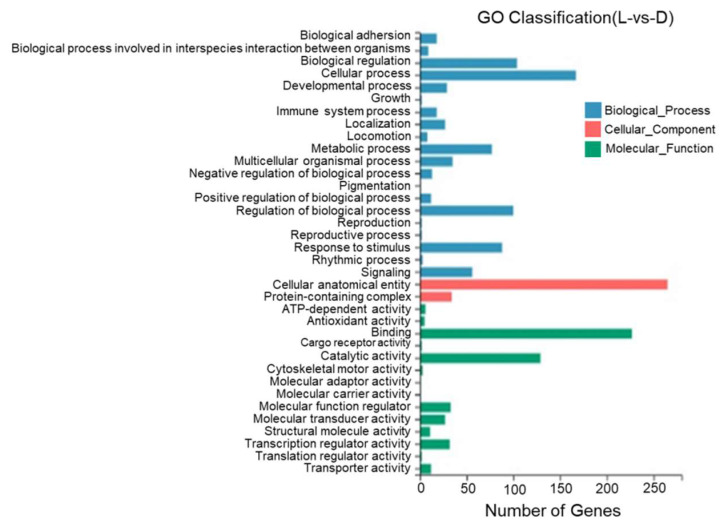
Gene Ontology functional classification of differentially expressed genes in the dorsal and lateral skin of *T. stenura*.

According to the results of a GO annotation analysis, 36 GO entries were identified between the dorsal and lateral skin with most DEGs being annotated to cell structures, binding, catalytic activity, the regulation of biological processes, stimulus response, and metabolic reactions. One DEG was annotated to pigment deposition (Figure 10).

**Figure 11:**
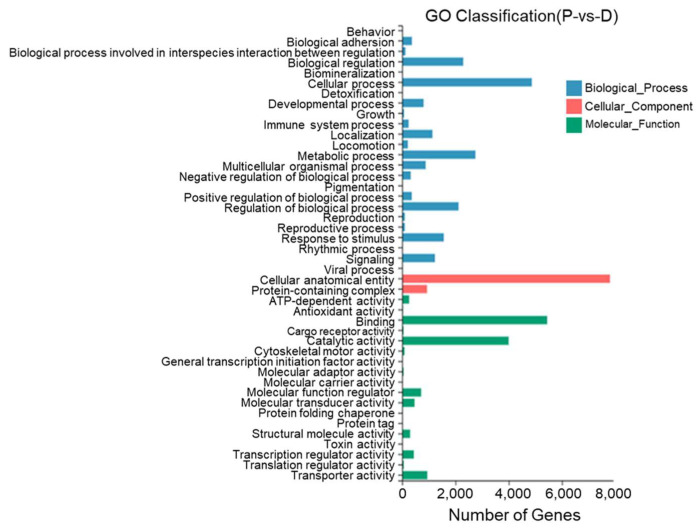
Gene Ontology functional classification of differentially expressed genes in the dorsal skin and peritoneal wall layer of *T. stenura*.

Between the dorsal skin and the peritoneal wall layer, 44 GO entries were identified with most DEGs annotated to cell structures, binding, cellular processes, catalytic activity, metabolic processes, the regulation of biological processes, and stimulus responses. Twenty-two DEGs were annotated to pigment deposition (Figure 11).

**Figure 12:**
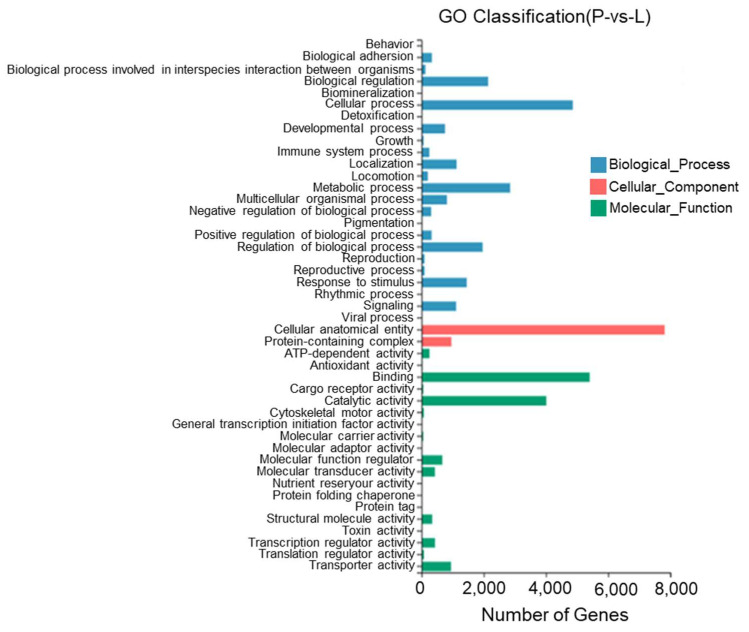
Gene Ontology functional classification of differentially expressed genes in the lateral skin and peritoneal wall layer of *T. stenura*.

According to the results, between the lateral skin and the peritoneal wall layer, 45 GO entries were identified with most DEGs being annotated to cell structures, binding, cellular processes, catalytic activity, metabolic processes, the regulation of biological processes, and stimulus responses. Twenty-three DEGs were annotated to pigment deposition (Figure 12).

### 3.5. Analysis of KEGG Enrichment Pathway for DEGs

**Figure 13:**
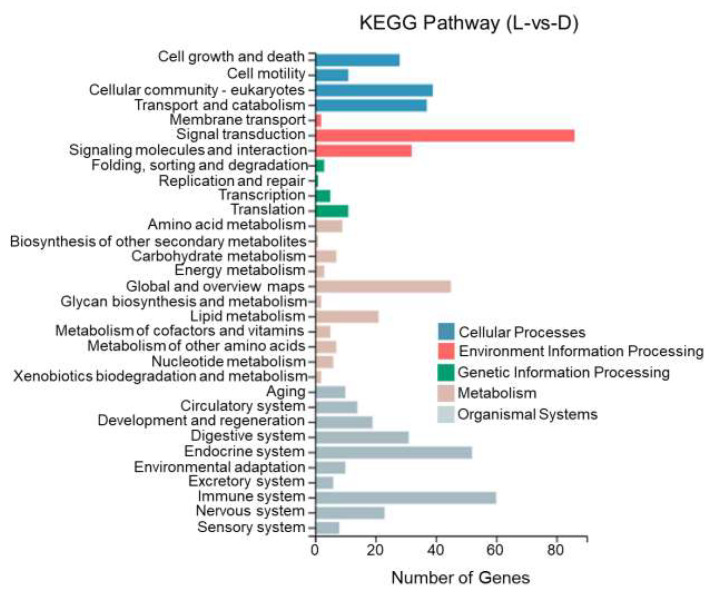
KEGG pathway classification of differentially expressed genes in the dorsal and lateral skin of *T. stenura*.

**Figure 14:**
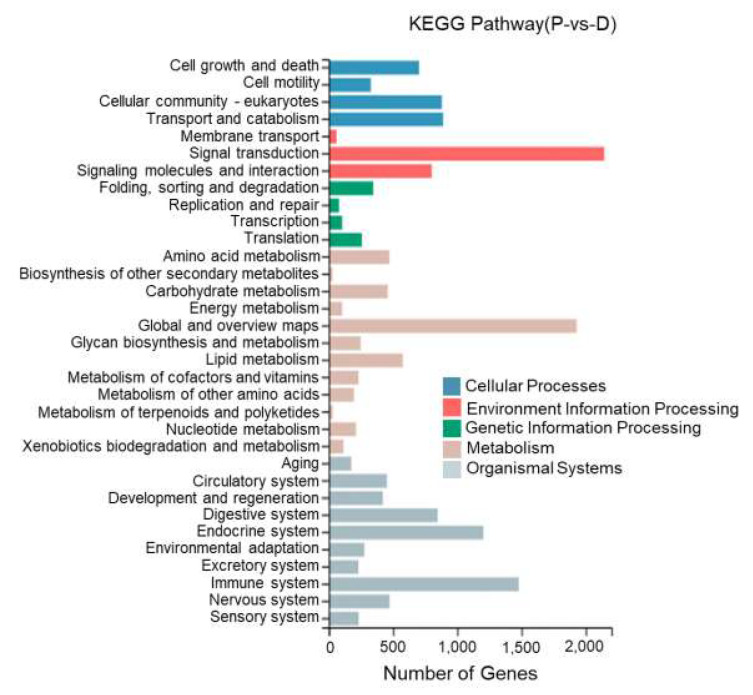
KEGG pathway classification of differentially expressed genes in the dorsal skin and peritoneal wall layer of *T. stenura*.

**Figure 15:**
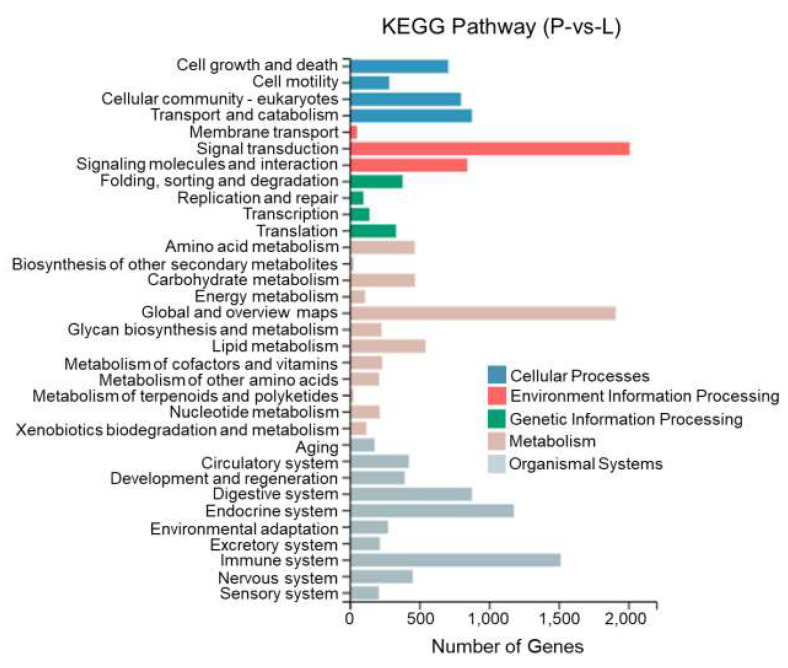
KEGG pathway classification of differentially expressed genes in the lateral skin and peritoneal wall layer of *T. stenura*.

This study performed KEGG enrichment analysis on DEGs between different tissues of *T. stenura* using a biological pathway classification. The results are shown in Figure 13, Figure 14 and Figure 15. The DEGs between the dorsal and lateral skin were enriched in 32 branches, the DEGs between the dorsal skin and the peritoneal wall layer were enriched in 33 branches, and the DEGs between the lateral skin and the peritoneal wall layer were enriched in 33 branches. These DEG-enriched branches mainly include cell growth and apoptosis, eukaryotic cell communities, transport and catabolism, signal transduction, signaling molecules and interactions, global and overview maps, the digestive system, the endocrine system, and the immune system. Most of the DEGs in these pathways were upregulated in the dorsal skin and the peritoneal wall layer.

Through a detailed biological pathway analysis of DEGs between different tissues of *T. stenura*, 22 pathways were enriched. These DEGs are mainly involved in metabolic pathways, the Rap1 signaling pathway, the Ras signaling pathway, the PI3K-Akt signaling pathway, the Wnt signaling pathway, the cAMP signaling pathway, the MAPK signaling pathway, the cell cycle, tyrosine metabolism, and melanin synthesis.

Most of the DEGs related to the MAPK signaling pathway, cAMP signaling pathway, cell cycle pathway, melanin synthesis, and tyrosine metabolism pathway were upregulated in the peritoneal wall layer. DEGs related to the Wnt signaling pathway, p53 signaling pathway, and ribosome pathway were upregulated in the dorsal skin. Some DEGs related to the Wnt signaling pathway and melanin synthesis pathway were upregulated in the lateral skin (Table 1, Table 2 and Table 3).

### 3.6. Screening Candidate Genes Related to the Melanin Synthesis Pathway

According to the transcriptome annotation results of different tissues of *T. stenura*, 23 DEGs were enriched in the melanogenesis pathway. These DEGs were identified by performing a Blast comparison of melanogenesis-related genes in the NCBI database (https://www.ncbi.nlm.nih.gov/gene/?term=Melanogenesis, accessed on 25 March 2022) with the melanogenesis pathway (https://www.kegg.jp/pathway/map04916, accessed on 25 March 2022). All three tissues had 23 DEGs enriched in the melanogenesis pathway (Table 4).

By downloading the RPKM values of these gene annotation analyses, it was found that compared to the lateral skin, the expression levels of *creb3* and *plcb* genes were significantly upregulated in the dorsal skin, while the expression levels of *asip* and *adcy2* genes were significantly downregulated. Compared to the peritoneal wall layer, the *creb3*, *gnao*, *map2k2*, *gnai*, *plcb*, and *camk2* gene expression levels were significantly upregulated in both the dorsal and lateral skin. The expression levels of *adcy9*, *creb1*, *wnt8*, *fzd2*, *dvl2*, *hras*, *raf1*, *edn1*, and *calm* genes were significantly downregulated in both the dorsal and lateral skin. Only the *wnt2* and *gsk3b* gene expression levels were significantly upregulated in the dorsal skin. The expression levels of the *asip*, *adcy2*, and *map2k1* genes were significantly downregulated in the dorsal skin. Only *tcf7* and *prkca* genes showed significant upregulation in the lateral skin. The expression level of the *kras* gene was significantly downregulated in the lateral skin.

### 3.7. Confirmation of the DEGs Identified with RNA-Seq by Quantitative Real-Time PCR

**Figure 16:**
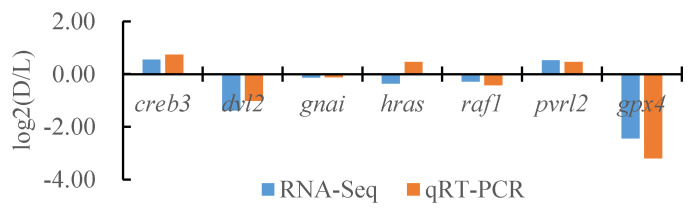
Comparison of the partial gene transcriptome results and qRT-PCR results in the dorsal skin and the lateral skin of *T. stenura*.

**Figure 17:**
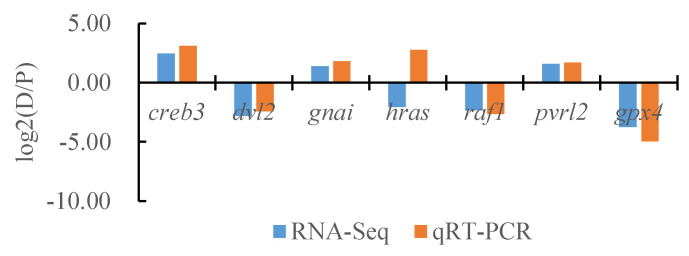
Comparison of the partial gene transcriptome results and qRT-PCR results in the dorsal skin and the peritoneal wall layer of *T. stenura*.

**Figure 18:**
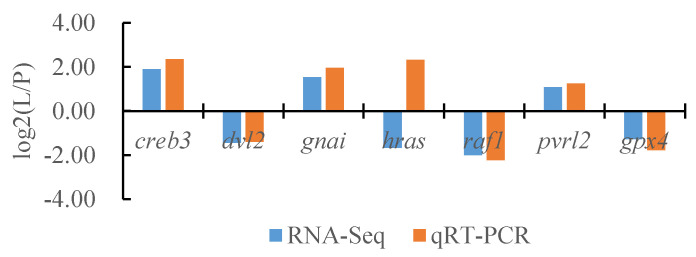
Comparison of the partial gene transcriptome results and qRT-PCR results in the lateral skin and the peritoneal wall layer of *T. stenura*.

To verify the reliability of the transcriptome data, we randomly selected seven DEGs and the internal reference gene α-*tublin* from RNA-seq and performed a quantitative analysis using qRT-PCR technology with three biological replicates per group. By comparing the transcriptome results of some genes between different tissues of *T. stenura* with the qRT-PCR results, we found that, except for the *hras* gene, the RNA-Seq and qRT-PCR analysis results of the other six DEGs were basically consistent (Figure 16, Figure 17 and Figure 18). The results show that the transcriptome data are reliable and can accurately detect DEGs in different tissues.

## 4. Discussion

### 4.1. Analysis of DEGs in Different Tissues of T. stenura

According to the results, most of the DEGs between the dorsal skin, the lateral skin, and the peritoneal wall layer of *T. stenura* were annotated to GO functional pathways such as cell structures, binding, cellular processes, catalytic activity, the regulation of biological processes, stimulus responses, and metabolic processes. Some DEGs were also annotated to the melanogenesis pathway. The functional annotations of the DEGs of three tissues suggested that changes in melanin synthesis were related to biological processes and molecular functional pathways. This was consistent with the study on the GO entries of pigment deposition in the skin and the peritoneal wall layer DEGs of three phenotypes of *Pristella maxillaris* [11]. In three different-colored skin tissues of *koi*, DEGs were found to be annotated to cell processes, single tissue processes, and metabolic processes in GO functional entries [22], indicating that body color regulation is related to metabolic pathways and cell proliferation and differentiation.

KEGG enrichment pathway analysis can identify the pathways regulated by DEGs, which is beneficial for further analysis of the molecular mechanisms of regulation. This study found that DEGs in the dorsal skin, the lateral skin, and the peritoneal wall layer of *T. stenura* were significantly enriched in processes such as the cell cycle, ribosome, melanin synthesis, tyrosine metabolism, the MAPK signaling pathway, the cAMP signaling pathway, the Wnt signaling pathway, and the PI3K-Akt signaling pathway. These pathways are closely related to melanin synthesis. Similar enrichment results were found in different skin tissues of other fish. Lin found that a large number of DEGs were enriched in the MAPK signaling pathway, the Wnt signaling pathway, and the tyrosinase metabolism process in a KEGG enrichment analysis of three different-colored skin tissues of *Scatophagus argus* [23]. Zhang found that DEGs in black tail and red abdominal skin tissues of *Lutjanus erythropterus* were enriched in the tyrosinase metabolism pathway, the melanin synthesis pathway, and the Wnt signaling pathway [24]. These upregulated genes may be involved in melanophore activity and melanin production processes.

In addition, DEGs in the skin of different developmental stages of *Carassius auratus* participated in the melanogenesis pathway and tyrosine metabolism process, and the genes in these pathways were closely related to body color changes [25]. The cAMP and MAPK signaling pathways are important pathways involved in melanin synthesis. cAMP, as a second messenger, activates related proteins to promote the expression of tyrosinase and produce more melanin. In studies of fish peritoneal tissue, Lu found that DEGs in the peritoneal wall layer of *S. younghusbandi* at different altitudes were enriched in the MAPK signaling pathway, the cAMP signaling pathway, and the melanogenesis process [15]. Ma found the same conclusion in the enrichment results of DEGs in the peritoneal wall layer of loaches from different altitude regions [26]. DEGs in different tissues of *T. stenura* were enriched in these pathways, which further illustrated that UV radiation factors can induce the synthesis of melanin, thereby promoting the adaptation of tissue organs to strong UV radiation environments.

### 4.2. Excavation of the Gene Related to Melanin Synthesis in Different Tissues of T. stenura

Melanin is the primary photoprotective substance that tissues and organs use to resist UVR damage. The degree of damage that UVR causes to the body depends on the amount of melanin [1]. Melanin synthesis is a multi-gene-regulated mechanism [27]. This study compared the transcriptome results of different tissues of *T. stenura* to the melanin synthesis pathway and found that nine DEGs were significantly upregulated in the peritoneal wall layer, while six DEGs were significantly upregulated in the dorsal and lateral skin. This indicated that the differences in melanin synthesis between different tissues were regulated by different genes, which jointly regulated melanin synthesis through signaling pathways such as α-MSH/MC1R, Wnt/β-catenin, Scf/c-Kit, and ET-1/ETB-R.

The α-MSH/MC1R signaling pathway is the most important regulatory pathway for fish melanin synthesis [28]. Studies had shown that the *asip* gene was an important inhibitory factor that regulated the synthesis of animal skin and hair melanin. It could block the effect of α-MSH on the MC1R receptor, inhibitd the expression of the MITF protein, and reduced the activity of tyrosinase and the synthesis of melanin in melanophores [29]. The overexpression of the asip gene in zebrafish could lead to the loss of melanophores in the back [30]. And, several genes that play key roles in pigmentation, i.e., *agouti*, *slc45a2*, *cbs*, *mift,* and *slc7a11*, showed significantly differential expression levels between brown and orange fish [31]. The expression of the *asip* gene in the dorsal skin of *T. stenura* was significantly reduced, while its expression in the lateral skin was reduced but not significantly. According to the phenotype, the melanin content of the dorsal skin was higher than that of the lateral skin, indicating that the synthesis of melanin in skin was related to the decreased expression of the *asip* gene. The expression of the *asip* gene in the peritoneal wall layer of *T. stenura* was not reduced, but its melanin content was higher than that of the dorsal skin. This may be due to an increase in the expression levels of other genes. Adenylate cyclase synthesized cAMP on the cell membrane and coordinated with the CREB binding protein to promote *mitf* gene transcription and produce melanin. The expression of the downstream genes, i.e., *adcy2*, *adcy9*, and *creb1* genes, was upregulated in the peritoneal wall layer of *T. stenura*, which promoted an increase in tyrosinase activity and thus led to the synthesis of more melanin [32,33].

The Wnt/β-catenin signaling pathway directly affects the proliferation and differentiation of melanophores [34]. The Wnt family genes *wnt2* and *wnt8* activate *fzd2*, which further activated the cytoplasmic disheveled proteins *dvl* and *dvl2*. These factors inhibited the activity of *gsk3b*, suppressed the phosphorylation and degradation of β-catenin, caused the accumulation of β-catenin in cells, activated the binding sites of *Lef* and *Mitf* transcription factors, and then activated *Mitf* expression [35]. In this study, the expression of *wnt2*, *wnt8*, *fzd2*, *gnao*, and *dvl2* genes in the peritoneal wall layer of *T. stenura* was significantly upregulated. This indicated that the Wnt/β-catenin signaling pathway played a key role in melanin synthesis in the peritoneal wall layer of *T. stenura*, which was consistent with Ma’s results on the KEGG enrichment of DEGs in the peritoneal tissue of loaches in different regions [26]. Similarly, in an KEGG enrichment analysis of DEGs in goldfish with three different skin colors, it was found that *wnt2* and *wnt11* genes were significantly upregulated [23].

The Scf/c-Kit signaling pathway plays an irreplaceable role in the proliferation, differentiation, and melanin synthesis of melanophores. When Scf binds to *c-kit*, it promotes the expression of the Ras protein, activates extracellular signal-regulated kinase (ERK), regulates the phosphorylation of the MITF protein, and promotes melanin synthesis [36]. The expression of the *kras*, *hras*, *raf1*, and *map2k1* genes in the peritoneal wall layer of *T. stenura* was upregulated, regulating the Scf/c-Kit signaling pathway to promote melanin production. In the skin and the peritoneal wall layer of *S. younghusbandi*, the DEGs *map3k4* and *map3k7* were significantly upregulated [15], indicating that melanin synthesis was related to the regulation of these genes. In addition, the *map2k2* gene was significantly upregulated in the dorsal and lateral skin of *T. stenura*, which may be related to the differences in the types of tissue that synthesize melanin, and there were differences in the molecular mechanisms that regulate melanin synthesis.

The binding of ET-1 with receptors is a key paracrine effect that promotes the proliferation of melanophores, regulates the *gnai* factor of G protein family members, activates phospholipase, and increases the expression of the *cal*, *camk2*, and *prkca* genes, thereby promoting melanin synthesis. The *edn1* and *cal* genes were significantly overexpressed in the peritoneal wall layer of *T. stenura*, while the *gnai*, *plcb*, and *camk2* genes were significantly overexpressed in the dorsal and lateral skin. In addition, the *prkca* gene was significantly overexpressed in the lateral skin, indicating that the ET-1/ETB-R signaling pathway was an important pathway for the skin and peritoneal wall layer to co regulate melanophore proliferation and melanin synthesis. Similarly, Fu et al. reported that the same pathway gene, i.e., *plcb* was involved in melanogenesis and evolved rapidly in *Nanorana parkeri* distributed in high elevations [37]. Bian et al. found that the *gnai* and *camk2* genes were significantly overexpressed in the skin and the peritoneal wall layer of wild-type *Pristella maxillaris* [11]. Lin found a correlation between *camk2* and pigmentation in the skin of *Scatophagus argus* [23]. Ma found that the *edn1* and *plcb* genes were significantly overexpressed and enriched in the melanin synthesis pathway through a local Blast comparison of transcriptome data from the alien species loach at high altitudes [26]. Overall, the regulatory factors of the ET-1/ETB-R signaling pathway were significantly overexpressed in the peritoneal wall layer and the dorsal skin of *T. stenura*, jointly promoting the synthesis of melanin in the tissue.

Melanin in fish plays an important role in resisting strong ultraviolet radiation. With the continuous deepening of relevant research, the understanding of melanophores and melanin resistance to UVR in high-altitude fish will become increasingly clear.

## 5. Conclusions

This study used the DNBSEQ platform to sequence and assemble the dorsal skin, the lateral skin, and the peritoneal wall of *Triplophysa stenura* by analyzing DEGs in different tissues and exploring genes related to the melanin synthesis pathway. The results indicate that after assembling and clustering, a total of 81,868 All Unigenes were obtained with a total length of 123,827,585 bp. A total of 68,750 Unigenes were annotated into seven functional databases. Most DEGs in the three tissues were annotated to GO entries related to the cell structure, binding, cellular processes, and catalytic activity, as well as KEGG pathways such as the cell cycle, the MAPK signaling pathway, the PI3K-Akt signaling pathway, the Wnt signaling pathway, tyrosine metabolism, and melanogenesis. By local Blast comparison, a total of twenty-three DEGs were shown to be enriched in the melanin synthesis pathway. Among them, nine DEGs were significantly upregulated in the peritoneal wall layer, and six DEGs were significantly upregulated in the dorsal and lateral skin. The results provide information that adds to our understanding of melanophores and melanin resistance to UVR in high-altitude fish.

## Figures and Tables

**Table 1:** KEGG functional analysis of DEGs in the dorsal skin and lateral skin of *T. stenura*.

Pathway (C-vs-B)	DEGs with Pathway	q-Value	Pathway ID
Metabolic pathways	44	2.6501 × 10^−23^	ko01100
Regulation of actin cytoskeleton	11	4.7186 × 10^−14^	ko04810
Melanogenesis	9	2.4110 × 10^−13^	ko04916
Vascular smooth muscle contraction	11	4.8707 × 10^−07^	ko04270
Wnt signaling pathway	9	4.8710 × 10^−07^	ko04310
Complement and coagulation cascades	14	1.0766 × 10^−06^	ko04610
cAMP signaling pathway	8	5.5857 × 10^−06^	ko04024
Purine metabolism	5	1.3700 × 10^−05^	ko00230
Rap1 signaling pathway	14	1.3687 × 10^−05^	ko04015
Calcium signaling pathway	13	1.3687 × 10^−05^	ko04020
Phospholipase D signaling pathway	11	1.3687 × 10^−05^	ko04072
Endocytosis	17	1.3687 × 10^−05^	ko04144
p53 signaling pathway	6	0.00010855	ko04115
PI3K-Akt signaling pathway	21	0.00012699	ko04151
MAPK signaling pathway	15	0.00052534	ko04010
Ras signaling pathway	12	0.00052534	ko04014
RNA transport	4	0.00095378	ko03013
Cell cycle	5	0.00175520	ko04110
Dopaminergic synapse	8	0.00778563	ko04728
Cytokine–cytokine receptor interaction	10	0.01653222	ko04060
Tyrosine metabolism	1	0.02147590	ko00350
Cytokine–cytokine receptor interaction	10	0.02484867	ko04060

**Table 2:** KEGG functional analysis of DEGs in the dorsal skin and peritoneal wall layer of *T. stenura*.

Pathway (M-vs-B)	DEGs with Pathway	q-Value	Pathway ID
Pyrimidine metabolism	80	1.3104 × 10^−09^	ko00240
Cardiac muscle contraction	156	3.0148 × 10^−08^	ko04260
MAPK signaling pathway	312	1.3427 × 10^−05^	ko04010
Ras signaling pathway	272	1.3427 × 10^−05^	ko04014
Cell cycle	69	2.0339 × 10^−05^	ko04110
Purine metabolism	173	7.3165 × 10^−05^	ko00230
Wnt signaling pathway	123	0.00044282	ko04310
Glycolysis/gluconeogenesis	80	0.00045747	ko00010
Cytokine–cytokine receptor interaction	160	0.00087131	ko04060
Rap1 signaling pathway	280	0.00167108	ko04015
Phospholipase D signaling pathway	251	0.00167108	ko04072
PI3K-Akt signaling pathway	448	0.00167108	ko04151
Complement and coagulation cascades	124	0.00167108	ko04610
Tight junction	274	0.00480370	ko04530
Melanogenesis	84	0.00503939	ko04916
Calcium signaling pathway	285	0.00705514	ko04020
cAMP signaling pathway	276	0.00705514	ko04024
p53 signaling pathway	85	0.00976914	ko04115
Ribosome	98	0.01100009	ko03010
Tyrosine metabolism	38	0.01262528	ko00350
DNA replication	31	0.01671852	ko03030
Nucleotide excision repair	27	0.01671852	ko03420

**Table 3:** KEGG functional analysis of DEGs in the lateral skin and peritoneal wall layer of *T. stenura*.

Pathway (M-vs-C)	DEGs with Pathway	q-Value	Pathway ID
Glycolysis/gluconeogenesis	86	0.027334015	ko00010
Purine metabolism	173	0.01563386	ko00230
Pyrimidine metabolism	89	4.65226 × 10^−10^	ko00240
Tyrosine metabolism	31	4.73656 × 10^−06^	ko00350
Glutathione metabolism	103	0.023045646	ko00480
Ribosome	148	0.043198018	ko03010
RNA transport	63	0.015772492	ko03013
DNA replication	38	0.005628575	ko03030
Nucleotide excision repair	32	0.005628575	ko03420
MAPK signaling pathway	276	0.000550616	ko04010
Ras signaling pathway	254	0.000550616	ko04014
Rap1 signaling pathway	243	0.000550616	ko04015
Calcium signaling pathway	277	0.027132289	ko04020
cAMP signaling pathway	265	0.027132289	ko04024
Cytokine–cytokine receptor interaction	179	3.94142 × 10^−07^	ko04060
Phospholipase D signaling pathway	229	5.24103 × 10^−05^	ko04072
Cell cycle	69	0.000365611	ko04110
p53 signaling pathway	89	0.027786679	ko04115
PI3K-Akt signaling pathway	437	0.000550616	ko04151
Vascular smooth muscle contraction	187	3.25907 × 10^−09^	ko04270
Wnt signaling pathway	102	0.029921916	ko04310
Melanogenesis	74	0.008839111	ko04916

**Table 4:** Candidate genes associated with melanin synthesis in different tissues of *T. stenura*.

Gene ID	Gene Symbol	Description	KEGG Orthology
Unigene16160_All	*asip*	agouti signaling protein	K08725
Unigene14361_All	*adcy2*	adenylate cyclase 2	K08042
Unigene16821_All	*adcy9*	adenylate cyclase 9	K08049
CL829.Contig2_All	*creb1*	cyclic AMP-responsive element-binding protein 1a	K05870
CL1198.Contig1_All	*creb3*	cyclic AMP-responsive element-binding protein 3	K09048
Unigene20918_All	*wnt2*	wingless-type MMTV integration site family, member 2	K00182
Unigene8674_All	*wnt8*	wingless-type MMTV integration site family, member 8	K00714
Unigene10110_All	*fzd2*	frizzled 2	K02375
Unigene23239_All	*gnao*	guanine nucleotide-binding protein G(o) subunit alpha	K04534
Unigene7220_All	*dvl2*	segment polarity protein dishevelled	K02353
Unigene13584_All	*gsk3b*	glycogen synthase kinase 3 beta	K03083
CL1735.Contig2_All	*tcf7*	transcription factor 7	K02620
CL5915.Contig5_All	*kras*	GTPase Kras	K07827
Unigene20200_All	*hras*	GTPase HRas	K02833
CL43.Contig11_All	*raf1*	RAF-1 proto-oncogene, serine/threonine-protein kinase	K04366
Unigene1090_All	*map2k1*	mitogen-activated protein kinase kinase 1	K04368
CL2442.Contig2_All	*map2k2*	mitogen-activated protein kinase kinase 2	K04369
CL9373.Contig1_All	*edn1*	endothelin-1	K16366
CL3664.Contig2_All	*gnai*	guanine nucleotide-binding protein G(i) subunit alpha	K04630
Unigene10095_All	*plcb*	phosphatidylinositol phospholipase C, beta	K05858
Unigene18911_All	*calm*	calmodulin	K02183
CL1057.Contig10_All	*camk2*	calcium/calmodulin-dependent protein kinase (CaM kinase) II	K04515
Unigene20135_All	*prkca*	classical protein kinase C alpha type	K02677

## Data Availability

The data presented in this study are available on request from the corresponding author. The data are not publicly available due to being part of a Master’s thesis.
